# Tetrahedral framework nucleic acid carrying angiogenic peptide prevents bisphosphonate-related osteonecrosis of the jaw by promoting angiogenesis

**DOI:** 10.1038/s41368-022-00171-7

**Published:** 2022-04-27

**Authors:** Dan Zhao, Dexuan Xiao, Mengting Liu, Jiajie Li, Shuanglin Peng, Qing He, Yue Sun, Jingang Xiao, Yunfeng Lin

**Affiliations:** 1 State Key Laboratory of Oral Diseases & National Clinical Research Center for Oral Diseases & Department of Oral and Maxillofacial Surgery, West China Hospital of Stomatology, Sichuan University, Chengdu, China; 2 Department of Oral and Maxillofacial Surgery, The Affiliated Stomatology Hospital of Southwest Medical University, Luzhou, China

**Keywords:** Oral diseases, Nanostructures

## Abstract

The significant clinical feature of bisphosphonate-related osteonecrosis of the jaw (BRONJ) is the exposure of the necrotic jaw. Other clinical manifestations include jaw pain, swelling, abscess, and skin fistula, which seriously affect the patients’ life, and there is no radical cure. Thus, new methods need to be found to prevent the occurrence of BRONJ. Here, a novel nanoparticle, tFNA-KLT, was successfully synthesized by us, in which the nanoparticle tetrahedral framework nucleic acid (tFNA) was used for carrying angiogenic peptide, KLT, and then further enhanced angiogenesis. TFNA-KLT possessed the same characteristics as tFNA, such as simple synthesis, stable structure, and good biocompatibility. Meanwhile, tFNA enhanced the stability of KLT and carried more KLT to interact with endothelial cells. First, it was confirmed that tFNA-KLT had the superior angiogenic ability to tFNA and KLT both in vitro and in vivo. Then we apply tFNA-KLT to the prevention of BRONJ. The results showed that tFNA-KLT can effectively prevent the occurrence of BRONJ by accelerating angiogenesis. In summary, the prepared novel nanoparticle, tFNA-KLT, was firstly synthesized by us. It was also firstly confirmed by us that tFNA-KLT significantly enhanced angiogenesis and can effectively prevent the occurrence of BRONJ by accelerating angiogenesis, thus providing a new avenue for the prevention of BRONJ and a new choice for therapeutic angiogenesis.

## Graphical abstract

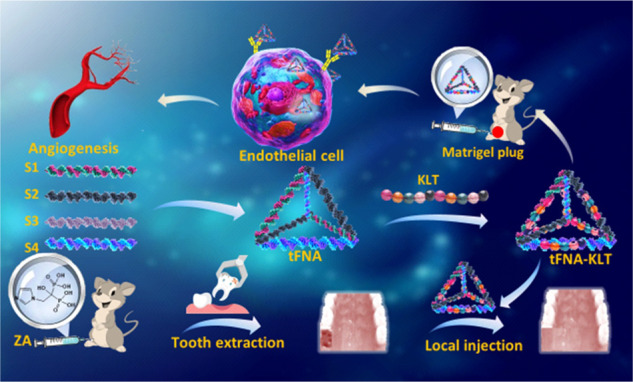

## Introduction

BRONJ is one of the medication-related osteonecrosis of the jaw (MRONJ).^1,2^ Patients have a history of using bisphosphonates to treat osteoporosis, tumor bone metastasis, and other diseases. Patients can have persistent pain, pus, limited mouth opening, long-lasting skin fistula and, even pathological fractures, which seriously affect the patients’ life, but there is still short of effective prevention and treatment measures in the medical community.^3,4^ At present, scholars have put forward a variety of hypotheses about the pathogenesis of BRONJ, mainly including inhibition of bone remodeling, reduction of angiogenesis, direct tissue toxicity, oral microbial infection, immunosuppression and so on, but its exact pathogenesis is still unclear.^5^

Previous reports have shown that bisphosphonates can inhibit the proliferative activity of endothelial cells (ECs) and the expression of angiogenic growth factors and then inhibit angiogenesis.^6–9^ Previous studies have shown that BRONJ can be treated by promoting angiogenesis.^10,11^ This shows that it is feasible to promote angiogenesis for the treatment of BRONJ. Therefore, we hope to prevent the occurrence of BRONJ by promoting angiogenesis when patients using bisphosphonates have to undergo necessary dental surgery, such as tooth extraction. There is no standardized BRONJ treatment scheme in the clinic. The principle is symptomatic treatment such as removal of dead bone and anti-inflammatory treatment.^12^ The prognosis of conservative treatment is poor.^13^ To effectively prevent patients from BRONJ after tooth extraction, clinical operations such as early wound closure after tooth extraction, hyperbaric oxygen therapy, stem cell therapy, and growth factor are mostly used, but there are few clinical data and it is difficult to reach a consensus.^14–17^ Therefore, at this stage, the clinic advocates prevention for BRONJ.

Angiogenesis is the primary requirement of wound healing. It can enhance the new vessels’ formation in the connective tissue of extraction sockets, promote soft tissue healing, therefore reducing the occurrence of BRONJ.^11^ On the other hand, the blood supply is conducive to osteogenesis. In the process of bone defect healing, angiogenesis occurs before osteogenesis, and angiogenesis and osteogenesis complement each other on bone regeneration.^18^ Then it can promote bone regeneration of tooth extraction wounds and reduce the occurrence of BRONJ. As for angiogenesis, the vascular endothelial growth factor (VEGF) is the major regulatory factor, which has a potent proangiogenic capacity.^19^ The high price, immunogenicity, and instability of VEGF limit its clinical application. KLT (Acetyl-KLTWQELYQLKYKGI-amide, also named QK) is a VEGF-mimic peptide, which can bind and then activate the VEGF receptor and play a function similar to VEGF.^20^ In addition, KLT has been proved that it can accelerate angiogenesis.^21–23^ Meanwhile, KLT has the advantages of low cost, low immunogenicity, easy synthesis, and good stability. Therefore, KLT becomes an alternative choice.

TFNA is a kind of DNA nanoparticle, which has attracted extensive attention in the biomedical field.^24–26^ Previous studies have found that tFNA can treat BRONJ by accelerating angiogenesis.^10^ Meanwhile, it has been reported that tFNA can deliver antimicrobial peptides to bacterial and tumor-penetrating peptides to breast cancer.^27,28^ New blood vessels or immature capillaries are necessary for homeostasis of tissue, local immunity, and regeneration or repairment of tissue, especially for the jaw with active metabolism and reconstruction.^11,18^ Therefore, in this study, we want to use tFNA for the delivery of the proangiogenic peptide KLT for further promoting angiogenesis, and then use the novel nanoparticle (tFNA-KLT) formed by tFNA and KLT for the prevention of BRONJ by advancing angiogenesis. As far as we know, we firstly studied the synergic effects of tFNA and proangiogenic peptides for accelerating angiogenesis and BRONJ prevention application.

## Results

### Synthesis, characterization, and cellular uptake of tFNA-KLT

**Fig. 1:**
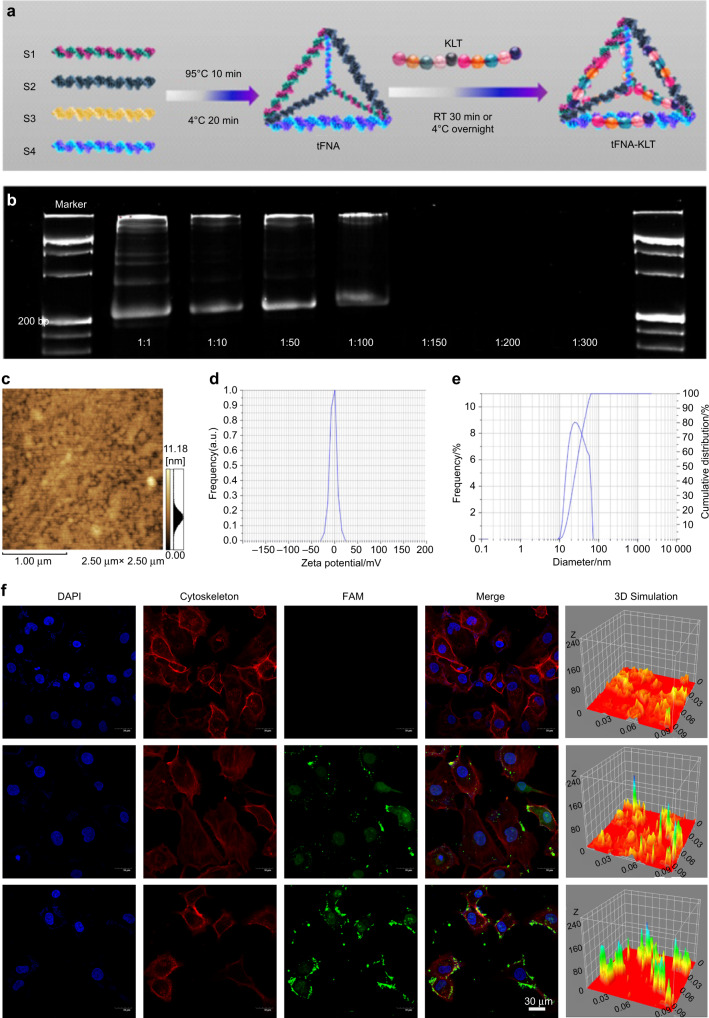
Preparation, characterization, and cellular uptake of tFNA-KLT. **a** Schematic diagram of the preparation of tFNA-KLT (RT room temperature). **b** Polypropylene Acyl Amine Gel Electrophoresis (PAGE) analysis of the successful synthesis of tFNA-KLT with the optimal ratio of tFNA and KLT. **c** Atomic force microscopy evaluation (scale bars are 1 µm). **d** Zeta-potential distribution of tFNA-KLT. **e** Size-distribution analysis of tFNA -KLT. **f** Reaction of FAM-modified KLT and tFNA-KLT with HUVECs by immunofluorescence staining (nucleus: blue; cytoskeleton: red; FAM: green). Scale bars are 30 µm

Figure 1a is a diagrammatic sketch showing the synthesis process of tFNA-KLT. In short, tFNA was firstly synthesized by four DNA single strands, and then tFNA and KLT were incubated at RT for 30 min or overnight at 4 °C in a certain ratio for the acquisition of tFNA-KLT. PAGE was used for determining the optimal ratio of tFNA and KLT combination. Since tFNA is negatively charged and KLT is positively charged, when tFNA-KLT is negatively charged, it indicates that the amount of KLT is still within the carrying range of tFNA and forms a band on PAGE. When tFNA are loaded with an appropriate amount of KLT, the formed tFNA-KLT has no charge and forms a band in the sample hole of PAGE. When it exceeds the carrying range of tFNA, tFNA-KLT were positively charged will not form bands on the PAGE. Based on the above principles and the results of Fig. 1b, the best combination ratio of tFNA and KLT was 1:100. Therefore, in the following experiment, the molecular ratio of tFNA to KLT in tFNA-KLT was 1:100. The results of AFM (Fig. 1c) showed the geometrical structure of tFNA-KLT. Meanwhile, the results of ζ distribution (Fig. 1d) showed that the ζ potential was about −2 mV. The particle size of tFNA-KLT (Fig. 1e) was about 15–30 nm. Immunofluorescence staining showed that KLT and tFNA-KLT were both can be taken in by HUVECs, and there was more tFNA-KLT interaction with HUVECs (Fig. 1f).

### tFNA-KLT promoted the proliferation and migration of HUVECs

**Fig. 2:**
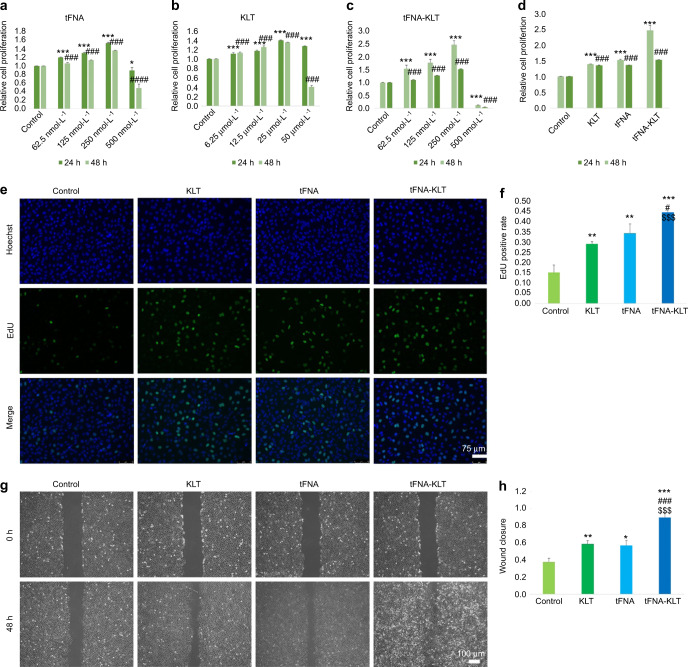
TFNA-KLT promoted the proliferation and migration of HUVECs. Cell counting kit-8 (CCK8) assay for the detection of effects of different concentrations of tFNA (**a**), KLT (**b**) and tFNA-KLT (**c**) on cell proliferation of HUVECs at 24 h and 48 h. (**d**) CCK8 assay of 250 nmol·L^−1^ ss-DNA, tFNA, and tFNA-KLT, and 25 μmol·L^−1^ KLT, on cell proliferation of HUVECs at 24 h and 48 h. Significant difference with respect to the control groups (*: 24 h; ^#^: 48 h), *^,#^*P* < 0.05, **^,##^*P* < 0.01, ***^,###^*P* < 0.001. **e** EdU assay of HUVECs (Hoechst: blue; EdU: green) and its quantification analysis (**f**). Scale bars are 75 µm. Wound-healing assay of HUVECs (**g**) and semi-quantification of the migration areas measured with ImageJ (**h**). Scale bars are 100 µm. Data are presented as means ± SD. Significant difference with respect to the control groups (*) or to tFNA group (^#^) or to KLT group (^$^), *^,#,$^*P* < 0.05, **^,##,$$^*P* < 0.01, ***^,###,$$$^*P* < 0.001

First, the effects of tFNA-KLT on HUVECs proliferative activity by the means of CCK8 and EdU assays were investigated. CCK8’s results showed that tFNA, KLT, tFNA-KLT can advance the proliferation of HUVECs in the concentration range of 250 nmol·L^−1^, 25 μM, 250 nM, respectively, and at this concentration, they showed the strongest ability to promote proliferation (Fig. 2a–c). In Fig. 2d, we compared the proliferation ability of each group at the optimal proliferation concentration. It showed that the group of tFNA-KLT had the strongest ability to promote the proliferation of HUVECs. Accordingly, we chose the concentration 250 nmol·L^−1^ (tFNA), 25 μmol·L^−1^ (KLT), 250 nM (tFNA-KLT, tFNA: KLT = 1:100), for the following experiments. The results of EdU assays were in accordance with that of CCK8 (Fig. 2e, f). To investigate the influences of tFNA-KLT on the migratory activities of HUVECs, the wound-healing assay was performed. TFNA-KLT significantly promoted the wound closure of the scratched HUVECs monolayers (Fig. 2g, h), compared with the other three groups. As we know, ECs’ proliferation and migration are the basis for angiogenesis. Therefore, in vitro, ex vivo, and in vivo angiogenesis assays were carried out.

### TFNA-KLT accelerated tube formation and spheroid sprouting

**Fig. 3:**
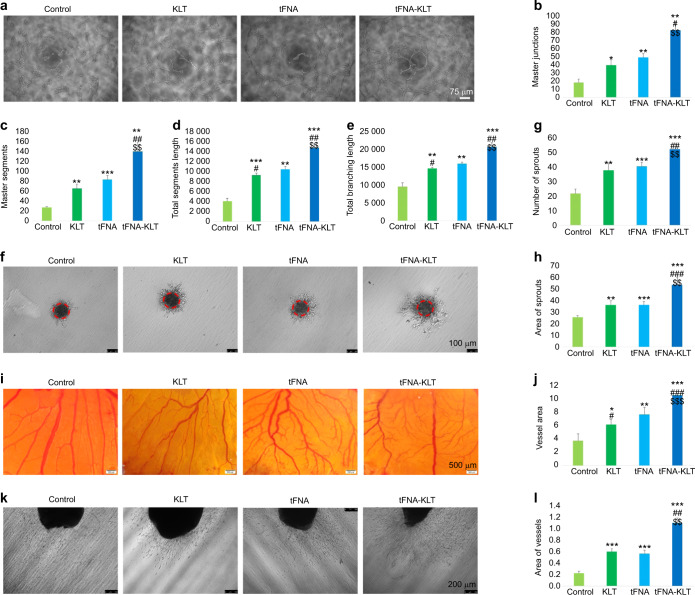
TFNA-KLT accelerated angiogenesis both in vitro and ex vivo. **a** Tube-formation assay for evaluating the angiogenic ability of the nanoparticles (ss-DNA, KLT, tFNA, and tFNA-KLT). Scale bars are 75 µm. Semi-quantification analysis of the master junctions (**b**), master segments (**c**), total segments length (**d**) and total branching length (**e**) of formed vessels by ImageJ. **f** Sprouting assay. Scale bars are 100 µm. Semi-quantification analysis of the number of sprouts (**g**) and area of sprouts (**h**). **i** Chick embryo chorioallantoic membrane (CAM) assay. Scale bars are 500 µm. **j** Semi-quantification analysis of the vessel area. **k** Aortic ring assay. Scale bars are 200 µm. **l** Semi-quantification analysis of the area of vessels. Data are presented as means ± SD. Significant difference with respect to the control groups (*) or to the tFNA group (^#^) or to KLT group (^$^), *^,#,$^*P* < 0.05, **^,##,$$^*P* < 0.01, ***^,###,$$$^*P* < 0.001

We then studied the effects of tFNA-KLT on the tube-formation capacity of HUVECs. Tube-like vessels can be observed on the Matrigel (Fig. 3a). Quantitative analysis was performed by ImageJ software and we found that tFNA-KLT significantly increased the number of master junctions and segments, total segment, and branching length in this tube-formation assay, compared with the group of control, KLT, and tFNA (Fig. 3b–e). Moreover, a spheroid-sprouting assay was also performed and vessels sprout from the edge of the spheroids (Fig. 3f). We demonstrated that tFNA-KLT can significantly increase the number and area of sprouts in the three-dimensional spheroid-sprouting assay (Fig. 3g, h), compared with the other three groups. The results of the tube-formation and spheroid-sprouting assay indicated that tFNA-KLT had enhanced ability in stimulating angiogenesis in vitro.

### TFNA-KLT stimulated angiogenesis both ex vivo and in vivo

**Fig. 4:**
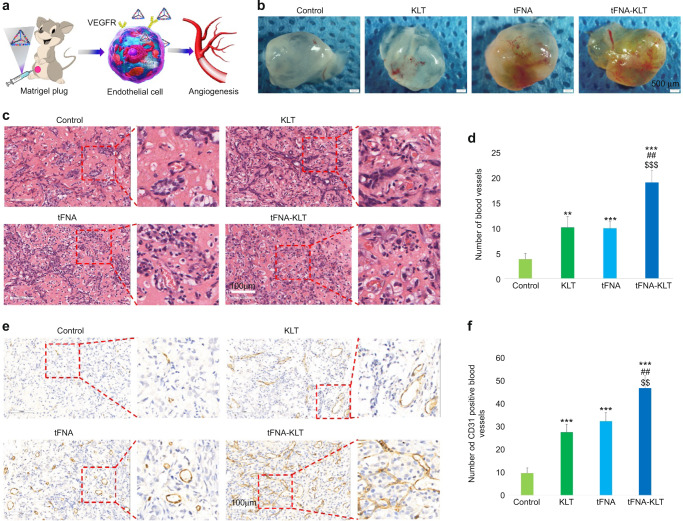
TFNA-KLT enhanced angiogenesis in vivo. **a** Schematic diagram of tFNA-KLT promoting angiogenesis in vivo. **b** Photographs of Matrigel plug assay after treated with nanoparticles (ss-DNA, KLT, tFNA, and tFNA-KLT). Scale bars are 500 µm. **c** HE staining of the gel and plenty of vessels can be seen. Scale bars are 100 µm. **d** Quantification analysis of the number of blood vessels with red blood cells in. **e** Immunohistochemistry of CD31. Scale bars are 100 µm. **f** Quantification analysis of the number of blood vessels with CD31 positive. Data are presented as means ± SD. Significant difference with respect to the control groups (*) or to tFNA group (^#^) or to KLT group (^$^), *^,#,$^*P* < 0.05, **^,##,$$^*P* < 0.01, ***^,###,$$$^*P* < 0.001

To further confirm proangiogenic results in vitro, we performed ex vivo and in vivo angiogenic assays. CAM’s results exhibited that the area of the branched vessels was larger in the group of KLT, tFNA, and tFNA-KLT, compared with the control group. Among them, tFNA-KLT demonstrated the strongest ability to promote angiogenesis (Fig. 3i, j). Meanwhile, neo-vessel outgrowth can be seen in the aortic ring assays and the results exhibited that the group of KLT, tFNA, and tFNA-KLT can significantly promote the sprout of vessels and the area of the vessels of them were also larger than that of the control group (Fig. 3k, l). TFNA-KLT showed more superior ability to stimulate angiogenesis. Then the Matrigel plug assay was carried out to study the influences of those nanoparticles on angiogenesis in vivo. It was as expected that many capillaries were formed in the plugs (Fig. 4b). From the HE-staining results, we can see a great many of blood vessels containing red blood cells (Fig. 4c). By quantitative image analysis, it exhibited that tFNA-KLT can notably increase the number of the vessels containing red blood cells, compared with control, KLT, and tFNA (Fig. 4d). Meanwhile, by CD31 staining, we confirmed the presence of ECs and the formation of neo-vessels (Fig. 4e). The results of quantitative image analysis showed that KLT, tFNA, tFNA-KLT can notably increase the quantity of CD31-positive blood vessels, compared with the control group (Fig. 4f). Compared with KLT and tFNA, tFNA-KLT presented a stronger proangiogenic ability. The results above indicated that KLT, tFNA, tFNA-KLT all can accelerate angiogenesis both ex vivo and in vivo. TFNA-KLT possessed a better ability to stimulate angiogenesis.

### TFNA-KLT prevented the occurrence of BRONJ

**Fig. 5:**
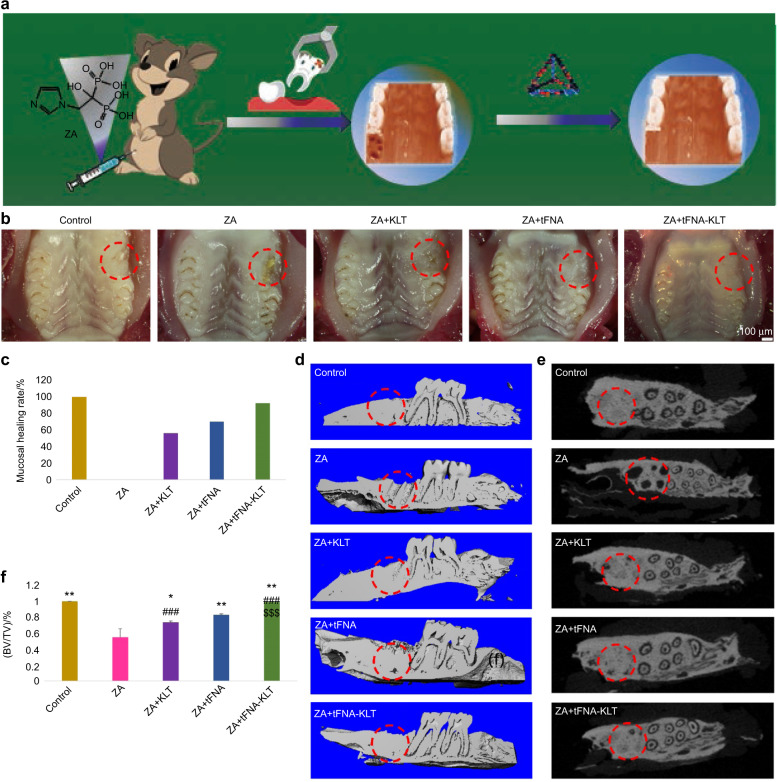
TFNA-KLT prevented the occurrence of Bisphosphonate-Related Osteonecrosis of the Jaw (BRONJ). **a** Schematic diagram of tFNA-KLT preventing BRONJ. **b** Healing of tooth extraction wound after 5 weeks of tooth extraction. Scale bars are 100 µm. **c** Mucosal healing rate. **d** 3D reconstruction of micro-CT (median sagittal section). **e** Results of micro-CT (transverse section). **f** Statistical results of BV/TV in different groups. Data are presented as means ± SD. Significant difference with respect to the control groups (*) or to the tFNA group (^#^) or to KLT group (^$^), *^,#,$^*P* < 0.05, **^,##,$$^*P* < 0.01, ***^,###,$$$^*P* < 0.001

Previous researchers have reported that bisphosphonates can inhibit ECs’ proliferation and downregulate angiogenesis-related growth factors, so as to reduce angiogenesis, which closely correlates with the occurrence of BRONJ.^6–9,29,30^ Therefore, we hope to prevent the occurrence of BRONJ by using nanoparticles that can promote angiogenesis, to cut down the incidence of BRONJ in patients who have used bisphosphonates after necessary dental operations, such as tooth extraction. As previous studies reported, tooth extraction is the most common cause of BRONJ. Thus, in this study, tooth extraction was used for the establishment of the BRONJ model. The healing of the tooth extraction socket was defined as no obvious extraction socket visible to the naked eye, complete mucosal coverage, no obvious inflammation, and no naked dead bone visible to the naked eye. This study’s results showed that the group of control and tFNA-KLT exhibited a high soft tissue healing rate of extraction socket, 100% and 92%, respectively (Fig. 5b, c). In the group of ZA, the dead bone was exposed obviously, and the tooth extraction wound was not covered by soft tissue. In the group of KLT and tFNA, some extraction sockets healed well, and some extraction sockets healed poorly, with a small amount of dead bone exposed. The soft tissue healing rate of them were 56 and 70%, respectively.

Bone regeneration within extraction sockets was three-dimensionally reconstructed and quantitatively analyzed by micro-CT. The group of control was fully repaired and the extraction sockets were filled with regenerated bone, as shown in Fig. 5d, e. The group of ZA was poorly healed and there was little new bone formation. Meanwhile, the formation of new bone can be observed in the group of KLT and tFNA. The extraction sockets were almost fully repaired in the group of tFNA-KLT. In addition, the micro-CT data was statistically analyzed. BV / TV is the volume fraction of bone tissue, which can reflect the amount of trabecular bone in different samples. BV/TV in the group of control, ZA, KLT, tFNA, and tFNA-KLT was 1.00, 0.55, 0.73, 0.83, and 0.99, respectively. This indicated that the quantitative results were consistent with the reconstruction data.

**Fig. 6:**
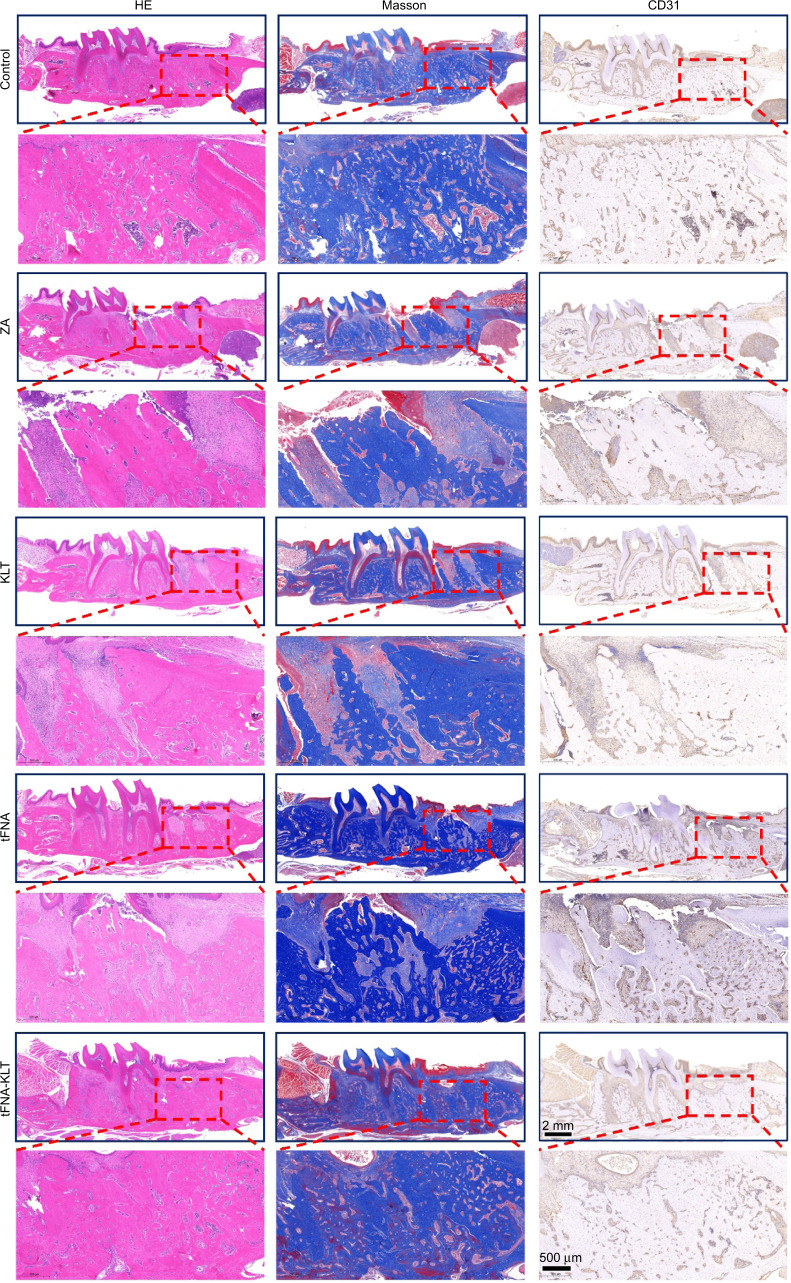
TFNA-KLT protected the rats from BRONJ by promoting angiogenesis. The results of H&E staining, Masson staining, and immunohistochemistry in the group of control, ZA, KLT, tFNA, tFNA-KLT. The group of control was fully repaired. However, the group of ZA was poorly repaired. Furthermore, the group of KLT and tFNA achieved partially healing of the extraction sockets. The group of tFNA-KLT was almost filled with new bone. Many blood vessels can be observed between the newly formed bone trabeculae in the extraction sockets in the group of tFNA-KLT

H&E and Masson trichrome staining were further used for the histological analysis of the formation of new bone. The results of them showed that the group of control was fully repaired (Fig. 6). However, the group of ZA was poorly repaired. Furthermore, the group of KLT and tFNA achieved partially healing of the extraction sockets. The superior outcome was confirmed in the group of tFNA-KLT, in which the extraction sockets were almost filled with new bone. Furthermore, the arrangement of cancellous bone and collagen fibers was more mature in the group of tFNA-KLT. Meanwhile, CD31 staining was further used for the detection of vessels in the extraction sockets. In the group of tFNA-KLT, many blood vessels can be observed between the newly formed bone trabeculae in the tooth extraction sockets. The results above showed the application of tFNA-KLT can effectively protect patients from BRONJ by promoting angiogenesis.

## Discussion

Previously, studies have shown that a peptide KLT, which mimicked VEGF helix 17–25, can induce VEGF-like biological activity response and KLT can accelerate angiogenesis, which also is verified in this study.^20–23^ Meanwhile, tFNA has also been reported that it can accelerate angiogenesis.^10,31–33^ Moreover, tFNA has successfully delivered antimicrobial peptides and tumor-penetrating peptides.^27,28^ Therefore, in view of these results we planned to use tFNA for the delivery of KLT and exploration of the potential therapeutic application of the new nanoparticle, tFNA-KLT, on vascular regeneration and further explore the potential application for the prevention of BRONJ associated with inhibition of angiogenesis. For achieving this goal, the functional properties of tFNA-KLT were extended by us. Angiogenesis is a complex biological process regulated by a variety of complementary and complex signals, which means several steps, especially stimulation endothelial cells proliferation, induction endothelial cell migration, and formation lumen-like structures by the stimulation of angiogenesis-related growth factors.^34^ To functionally characterize the biological activity of tFNA-KLT, the effects of tFNA- KLT on the basic functions of ECs, such as proliferation, migration, and lumen formation, were evaluated. In this study, we proved that tFNA-KLT can accelerate the proliferation, migration, and lumen formation of ECs, which was stronger than that of KLT and tFNA. In the following series of angiogenesis experiments in vitro, ex vivo, and in vivo, we also proved that tFNA-KLT had an enhanced ability to facilitate angiogenesis, compared with KLT and tFNA.

TFNA has exhibited to be an excellent carrier, which has attracted considerable attention from many scholars. It has been reported to carry aptamers, miRNA, antisense peptide nucleic acids, cationic polymers, antimicrobial peptides, and so on and showed a wide application potential in the fields of biomedical applications.^35–40^ Therefore, in this study, we modified tFNA with a proangiogenic peptide, KLT, hoping for the enhancement of angiogenesis. Meanwhile, tFNA has been reported that it possesses good biological activity, which can enhance the proliferative, migratory, and tube-formation abilities of ECs and promote the healing of diabetic wounds and cure of BRONJ by accelerating angiogenesis.^10,32^ Previous study has found that tFNA can increase the serum stability of peptides.^28^ In this study, on the one hand, tFNA played its role in promoting angiogenesis. On the other hand, as a carrier, tFNA enhanced the stability of KLT and carried more KLT to interact with endothelial cells and enhanced angiogenesis. In general, the newly formed nanoparticle tFNA-KLT displayed a strong ability to accelerate angiogenesis and had the potential to be applied to tissue engineering vascularization and other ischemic diseases.

For patients who have a history of bisphosphonates treatment, it is particularly important to take necessary measures to prevent BRONJ after tooth extraction or other alveolar surgery. Bisphosphonates are reported that they can inhibit angiogenesis and then cause BRONJ. Previous researchers have reported that tFNA can facilitate the healing of BRONJ and diabetic wound by boosting angiogenesis.^10,32^ Because there is no ideal treatment scheme for BRONJ at present, its prevention is more important than treatment. Therefore, in this study, we applied tFNA-KLT to the prevention of BRONJ. From the histology, micro-CT, and immunohistochemically staining results, tFNA-KLT can promote the formation of new bone and effectively prevent the occurrence of BRONJ by promoting angiogenesis. Studies have shown that hyperbaric oxygen therapy can increase the effective oxygen content of tissue, promote the proliferation of capillaries, accelerate the formation of collateral circulation, and then promote osteogenesis, so as to accelerate the repair and healing of lesions in BRONJ patients.^41,42^ Platelet-rich plasma (PRP) has been clinically used for the prevention of BRONJ. PRP contains a large number of growth factors, which can reduce inflammation, accelerate angiogenesis, and therefore promote tissue healing.^43–45^ All these show that promoting angiogenesis can effectively treat and prevent the occurrence of BRONJ, which also provides theoretical support for the results of this study. Some anticancer drugs mainly targeting VEGF or VEGF-related pathways, such as bevacizumab and sunitinib, can inhibit angiogenesis, and their use will also lead to MRONJ.^46–49^ According to our results, tFNA- KLT has the potential not only to be applied to BRONJ but also to this kind of MRONJ.

Patients who have cancer, they use ZA to inhibit tumor bone metastasis, treat bone damage of multiple myeloma and hypercalcemia. If the use of other drugs affects their function in the whole body, it may lead to tumor metastasis, bone damage, and hypercalcemia, which may threaten the lives of patients. Therefore, in order not to affect the therapeutic effect of ZA, we used local injection of tFNA-KLT into the tooth extraction socket to prevent the occurrence of BRONJ.

In summary, a novel nanoparticle, tFNA-KLT, was successfully synthesized by us. First, it was confirmed that tFNA-KLT had the superior angiogenic ability to tFNA and KLT both in vitro and in vivo. To further verify the application of tFNA-KLT in disease model, then we apply tFNA-KLT to the prevention of BRONJ. The results showed that tFNA-KLT can effectively prevent the occurrence of BRONJ by facilitating angiogenesis. In addition, tFNA exhibited superior peptide carrying performance and may be an advantageous carrier for carrying multiple peptides. The newly formed nanoparticle, tFNA-KLT, possessed the same characteristics as tFNA, such as simple synthesis, stable structure, and good biocompatibility. Meanwhile, tFNA enhanced the stability of KLT and carried more KLT to interact with ECs. In addition, small proangiogenic molecules such as aptamers and miRNAs can also be connected to the top or side arms of tFNA-KLT to further improve the proangiogenic ability of tFNA-KLT. All in all, the prepared novel nanoparticle significantly enhanced angiogenesis and can effectively prevent the occurrence of BRONJ by accelerating angiogenesis, thus providing a new avenue for the prevention of BRONJ and a new choice for therapeutic angiogenesis.

## Materials and methods

### Fabrication and characterization of tFNA-KLT

As reported, tFNA were synthesized by mixing equimolar concentrations of four DNA single strands (ss-DNA) (Table 1 showed the sequences of these ss-DNA) in the TM buffer (consisting of 10 nM Tris-HCl and 50 nM MgCl_2_).^35,36^ (95 °C for 10 min, 4 °C for 20 min).

**Table 1:** Base sequence of each ss-DNA

ss-DNA	Base sequence (5′-3′)
S1	ATTTATCACCCGCCATAGTAGACGTATCACCAGGCAGTTGAGACGAACATTCCTAAGTCTGAA
S2	ACATGCGAGGGTCCAATACCGACGATTACAGCTTGCTACACGATTCAGACTTAGGAATGTTCG
S3	ACTACTATGGCGGGTGATAAAACGTGTAGCAAGCTGTAATCGACGGGAAGAGCATGCCCATCC
S4	ACGGTATTGGACCCTCGCATGACTCAACTGCCTGGTGATACGAGGATGGGCATGCTCTTCCCG

TFNA is self-assembled from negatively charged DNA and tFNA is therefore also negatively charged, which is also confirmed by previous research.^37^ KLT is positively charged. Therefore, tFNA and KLT can be combined by electrostatic adsorption. To form tFNA-KLT, tFNA and KLT were incubated at room temperature (RT) for 30 min or overnight at 4 °C in a certain ratio. Figure 1a is a schematic diagram of the synthesis process. To determine the optimal ratio of tFNA and KLT combination, polyacrylamide gel electrophoresis (PAGE) was performed. ζ potential and particle size analyzer were used for the detection of ζ potential and size of tFNA-KLT. Atomic force microscopy (AFM) was performed for shape reflection of tFNA-KLT.

### Cellular uptake of tFNA-KLT

Cellular immunofluorescence staining was performed to confirm whether human umbilical vein ECs (HUVECs) can take up *tFNA-KLT*. The addition of FAM (6-carboxyfluorescein) modified KLT and tFNA-KLT to the culture medium (the concentration was 250 nM), and then interacted for 6 h. To capture images, HUVECs were fixed with 4% paraformaldehyde. The cytoskeleton was stained by phalloidin and the nucleus was stained by DAPI.

### Proliferation assay

For evaluation of the influences of tFNA, KLT and tFNA-KLT on HUVECs proliferation, cell counting kit-8 (CCK8) assay and 5-Ethynyl-2′-deoxyuridine(EdU) assay were carried out. For CCK8, inoculate HUVEC (1 × 10^4^) in a 96-well plate overnight for attachment. After 24 h of starvation, replace the medium with serum-free medium containing 62.5 nmol·L^−1^, 125 nmol·L^−1^, 250 nmol·L^−1^, 500 nmol·L^−1^ tFNA and tFNA-KLT (tFNA: KLT = 1:100), 6.25 μmol·L^−1^, 12.5 μmol·L^−1^, 25 μmol·L^−1^, 50 μmol·L^−1^ KLT, respectively. To further confirm the influences of tFNA, tFNA-KLT, and KLT on the proliferation of HUVECs, EdU assay (US Everbright^®^ Inc, USA) was carried out by following the instructions.

### Cellular migration assay

ECs’ migration is a cardinal step for angiogenesis. Therefore, to detect the influences of tFNA, tFNA-KLT, and KLT on migratory activities of HUVECs, a wound-healing assay was carried out. After 24 h, scrape off by using the tip of a sterile pipette and form a two-way wound. Then, 250 nmol·L^−1^ of tFNA and tFNA-KLT (tFNA: KLT = 1:100), 25 μmol·L^−1^ of KLT, respectively, were added to the groups. After 0 h and 48 h incubation, wound closures pictures were taken, respectively. The wound closure area was measured by Image J.

### Tube-formation assay

Tube-formation assay was carried out for assessing the influences of tFNA, tFNA-KLT, and KLT on angiogenic ability in vitro. Addition of 250 nmol·L^−1^ tFNA and tFNA-KLT (tFNA: KLT = 1:100), 25 μmol·L^−1^ KLT, respectively to the culture medium. Twenty-four hours later, 100 μL of HUVECs (1–1.5 × 10^5^ per mL) were inoculated to 96-well plates covered with Matrigel. The pictures of tubular formation were taken at 6 h and analyzed by ImageJ.

### Spheroid-sprouting assay

Add 1.5% agarose to 96-well plates (50 μL per well) and sterilize with ultraviolet light. Then add 100 μL of HUVECs (1 × 10^3^ per well) to the above plates, and harvest spheroids after 72 h. Next, cover 96-well plates with Matrigel and move spheroids into them. The medium had 250 nmol·L^−1^ tFNA and tFNA-KLT (tFNA: KLT = 1:100), 25 μmol·L^−1^ KLT, respectively. Observe and photograph the sprouts of spheroids after 48 h.

### Aortic ring assay

Aortas isolated from two-week-old C57 female mice were cut into approximately 1 mm long rings. The aortic rings then were placed in 96-well plates and covered with 50 μL rat tail collagen gel. After incubation for 15 min allowing for rat tail collagen gel polymerization, 100 μL culture medium contained 250 nmol·L^−1^ tFNA and tFNA-KLT (tFNA: KLT = 1:100), 25 μmol·L^−1^ KLT, respectively, were added into the wells. The aortic rings were photographed after 72 h incubation and the area of vascular sprouting from the aortic rings was measured using ImageJ software.

### Chick embryo chorioallantoic membrane (CAM) assay

Hatch the fertilized sterile eggs in an incubator at 37 °C and 60% humidity for 7 days, and next the windows were opened. Then put a gelatine sponge on the CAM, and add 100 μL of 250 nmol·L^−1^ tFNA and tFNA-KLT (tFNA: KLT = 1:100), 25 μmol·L^−1^ KLT, respectively, to the gelatine sponge, and then close the window. After incubation for 72 h, take out the gelatine sponge and take pictures of vessels on the CAM by a stereomicroscope. The pictures were analyzed by using ImageJ software.

### Matrigel plug assay

To detect the angiogenic abilities of tFNA, tFNA-KLT, and KLT in vivo, Matrigel plug assays were performed. Randomly divide nude mice into 4 groups (female, 5-week-old BALB/c, four mice per group). First, 100 μL HUVECs (1 × 10^6^) and 400 μL Matrigel were mixed. Inject the mixture subcutaneously into the right ventral side of the mice. 100 μL of TM buffer, 1 mmol·L^−1^ tFNA and 1 mmol·L^−1^ tFNA-KLT (tFNA: KLT = 1:100), 100mmol·L^−1^ KLT, respectively, were daily locally injected into Matrigel plugs for a week. Figure 4a is the schematic diagram of the Matrigel plug assay. After 7 days, the Matrigel plugs were removed for CD31and hematoxylin and eosin (H&E) staining.

### Animal treatment and surgery

There were five groups: control, ZA, ZA + tFNA, ZA + KLT, and ZA + tFNA-KLT. Altogether 50 SD rats (male, 8 weeks old, 180–200 g) were randomly separated. Dexamethasone (5 mg·kg^−1^) was injected intraperitoneally weekly for 5 weeks. Except for the control group, other rats received intravenous Zoledronic acid (ZA) (125 μg·kg^−1^) for 5 weeks (twice per week). Extract the rat’s left maxillary first molar at the fifth weekend. The ZA + tFNA group received 100 μL of tFNA (1 mmol·L^−1^) daily for 1 week after surgery at local mucosa. Simultaneously, the ZA + KLT group received 100 μL of KLT (100 mM). The ZA + tFNA-KLT group received 100 μl of tFNA-KLT (tFNA: KLT = 1:100) (1 mmol·L^−1^). The control group and ZA group were received with the same amount of saline for 5 weeks. Five weeks later, the rats were euthanized and the maxilla was obtained to continue the subsequent detection and analysis. Figure 5a is the schematic diagram of this assay. Animal experiments were conducted under the guidance of the Animal Commission of Sichuan University.

### Micro-computed tomography (Micro-CT) analysis

To observe the healing of the jaw bone from the extraction wound, Micro-CT was used for scanning the maxillary bone. Scan the left maxilla of the rat with micro-CT (μCT50, SCANCO Medical AG, Switzerland) (10 μm, 70 kVp, 200 μA, 1 × 300 ms). SCANCO Medical Evaluation software was used for scanned data evaluation and reconstruction. The tooth extraction wound was drawn as the region of interest (ROI) for bone volume (BV) measurement. The ratio of BV to tissue volume (TV) of the ROI was measured for the quantitative analysis by Micro-CT evaluation program. Then the tissue blocks were taken for H&E staining, Masson staining, and tissue immunofluorescent staining.

### Statistical analysis

The mean values of each group were compared by two-tailed *t* test or one-way ANOVA. *P* value was <0.05 (*, ^#^, ^$^), meaning statistical significance.
